# Frailty state among Indonesian elderly: prevalence, associated factors, and frailty state transition

**DOI:** 10.1186/s12877-019-1198-8

**Published:** 2019-07-03

**Authors:** Siti Setiati, Purwita Wijaya Laksmi, I.G.P. Suka Aryana, Sri Sunarti, Novira Widajanti, Lazuardhi Dwipa, Euphemia Seto, Rahmi Istanti, Laurentius Johan Ardian, Sabrina Chusnul Chotimah

**Affiliations:** 1 Division of Geriatric, Department of Internal Medicine, Faculty of Medicine Universitas Indonesia, Cipto Mangunkusumo Hospital, Jakarta, Indonesia; 2 Clinical Epidemiology and Evidence Based Medicine Unit, Faculty of Medicine Universitas Indonesia, Cipto Mangunkusumo Hospital, Jalan Pangeran Diponegoro No. 71, Jakarta, 10430 Indonesia; 3 Division of Geriatric, Departement of Internal Medicine, Faculty of Medicine, Universitas Udayana, Sanglah Teaching Hospital, Bali, Bali Indonesia; 4 Division of Geriatric, Department of Internal Medicine, Faculty of Medicine, Universitas Brawijaya, dr. Syaiful Anwar Hospital, Malang, East Java Indonesia; 5 Division of Geriatric, Departement of Internal Medicine, Faculty of Medicine, Universitas Airlangga, Dr. Soetomo General Hospital, Surabaya, East Java Indonesia; 6 Division of Geriatric,Department of Internal Medicine, Faculty of Medicine, Universitas Padjajaran, Hasan Sadikin General Hospital, Bandung, West Java Indonesia

**Keywords:** Frailty, Frailty transition, Risk factors, Prognostic factors, Prognostic score, Elderly

## Abstract

**Background:**

Information about frailty status and its transition is important to inform clinical decisions. Predicting frailty transition is beneficial for its prevention. While Indonesia is the 4th largest geriatric population in Asia, data about frailty transition is limited. This study aimed to obtain data on prevalence of frailty, its risk factors, frailty state transition and its prognostic factors, as well as to develop prognostic score for frailty state transition.

**Methods:**

Multicenter study on subjects aged ≥60 years old was done to obtain the prevalence of frailty status and to identify risk factors of frailty. Prospective cohort over 12 months was done to obtain data on frailty state transition. Multiple logistic regression analysis was performed to identify its prognostic factors from several clinical data, which then were utilized to develop prognostic score for frailty state worsening.

**Results:**

Cross-sectional data from 448 subjects showed that 25.2% of the subjects were frail based on Frailty index-40 items. Risk factors of frailty were age (OR 2.72; 95% CI 1.58–4.76), functional status (OR 2.89; 95% CI 1.79–4.67), and nutritional status (OR 3.75; 95% CI 2.29–6.13). Data from the 162 subjects who completed the cohort showed 27.2% of the cohort had frailty state worsening. Prognostic factors for frailty state worsening were being 70 years or older (OR 3.9; 95% CI 1.2–12.3, *p* < 0.05), negative QoL, i.e., fair and poor QoL (OR 2.5; 95% CI 1.1–5.9, *p* < 0.05), and slow gait speed (OR 2.8; 95% CI 1.3–6.4, *p* < 0.05). The internal validation of the prognostic score consisted of those three variables showed good performance.

**Conclusion:**

The prevalence of frailty in this study among Indonesian elderly in outpatient setting was 25.2%. The risk factors of frailty were age, functional status and nutritional status. The prognostic factors for frailty state worsening were being 70 years old or older, negative QoL (fair or poor quality of life), and slow gait speed. A prognostic score to predict frailty state worsening in 12 months had been developed.

## Background

Indonesia is home to the eighth largest population of older persons in the world and the fourth largest among Asian countries. With around 21 million older persons (8.2%) in the population, Indonesia presents the largest number of older persons in Southeast Asia [1]. Frailty has become a major health problem among Indonesian elderly. Frailty is defined as a state of excess vulnerability to stressor due to age-related decline in physiologic reserve throughout multiple organ systems, resulting in inadequacy to preserve or recover homeostasis after a destabilization [2]. Knowledge about the effect of frailty on important health-related outcomes is well-established. Frailty has been recognized as increasing the risk of important health outcomes in older persons such as falls, hospitalization, disability, poor health-related quality of life (QoL), and mortality [3–5].

Frailty is a spectrum ranging from lesser to greater frailty states. It is classified into fit or robust, prefrailty, and frailty. Frailty is a dynamic process. It is possible for an individual to experience frailty state transition over time. The frailty state transition can be a frailty state improvement into a lesser frailty state or a frailty state worsening [6, 7]. Frailty state worsening is more common than frailty state transition into a lesser frailty state [6, 8]. Several studies have identified various factors associated with frailty transition in community-dwelling older adults, which includes baseline frailty state, the presence of certain diseases (e.g., diabetes mellitus, cardiovascular disease, and cancer), low functional status, as well as age itself [6, 9]. However, there are only a limited number of longitudinal studies on the frailty state transition in the Asian population, who may have different risk factors in relation to their own characteristics.

This study aimed to obtain the prevalence of frailty status among Indonesian elderly and its risk factors. The study also aimed to obtain data on frailty state transition over a 12-month period and its prognostic factors. Lastly, the study aimed to develop a practical scoring system to predict frailty state worsening in 12 months from the prognostic factors.

## Methods

### Study design and subjects

This study consisted of a multicenter cross-sectional study and a 12-month prospective cohort. A cross-sectional study was performed to obtain the prevalence of frailty status and its risk factors among Indonesian elderly. A prospective cohort was used to obtain data on the frailty state transition and the prognostic factors for frailty state worsening, as well as to develop a prognostic scoring system for frailty state worsening. The subjects were recruited from hospitals which provide health care service for the elderly. Health care service for elderly patients in Indonesia has developed rapidly since the government issued a regulation in 2014 regarding elderly patients’ services in hospitals. The regulation arranged for each hospital to have special elderly patients’ services. Elderly in Indonesia are defined as those aged 60 years and above. There have been 15 government hospitals that provide elderly patients’ services in Indonesia in accordance with the rules contained in the regulation. These hospitals are Regional Referral Hospitals and one of them is a National Referral Hospital. In this study, we randomly selected 5 out of those 15 referral hospitals for data collection of the cross-sectional study, namely, Dr. Soetomo Hospital which is the referral hospital for East Java Province, Sanglah Hospital, which is the referral hospital in Bali Island, Hasan Sadikin Hospital, which is the referral hospital for West Java Province, Dr. Saiful Anwar Hospital, which is the referral hospital for Malang Region, and Cipto Mangunkusumo Hospital (RSCM), which is the National Referral Hospital. Patients receiving elderly patients’ services at these hospitals are those referred by primary health care and smaller hospitals. RSCM is the national referral hospital, receiving a referral from regional hospitals. Subjects were recruited consecutively for 2 months in all selected hospitals. Half of the subjects were recruited from RSCM, while the rest were recruited from Regional Referral Hospitals. The inclusion criteria for cross sectional study were all patients aged 60 years or above who visited the geriatric outpatient clinic and agreed to join and be assessed for the study. The inclusion criteria for prospective cohort study were all patients aged 60 years or above who visited the geriatric outpatient clinic and agreed be assessed for baseline data and the follow up in 1 year. Exclusion criteria included cognitive impairment (i.e., a score of Abbreviated Mental Test ≤7), acute diseases (e.g., severe infection, acute cerebrovascular events, acute cardiovascular events), and refusal to participate in the study. The sample size for the cross-sectional study was determined based on the equation for the sample size of the estimated proportion. The minimum number of subjects to be recruited was 441 subjects. Some of the subjects in the cross-sectional study were selected for the prospective cohort over 12 months. For the cohort study, we selected the National Referral Hospital, RSCM, as the center for the study due to its clinical and demographic diversity, which may represent the Indonesian population in general. The sample size for the cohort was determined based on estimated relative risk with 5% alpha and 80% power. Follow-up was to be done with a minimum of 170 subjects. Loss of contact in the 12th-month follow-up resulted in a dropout from the study. Ethical approval was obtained from the Faculty of Medicine Universitas Indonesia. Written informed consent was obtained from the subjects or their representing family members.

### Baseline data collection

This study mostly used primary data obtained using questionnaires, only data about comorbidities were collected from medical records. Data collected for the cross-sectional study included (a) demographic data (i.e., age and sex); (b) frailty state based on the Frailty Index-40 item (FI-40) questionnaire; (c) functional status based on Barthel Index of Activity of Daily Living (ADL) questionnaire: totally dependent (score 0–4), heavily dependent (score 5–8), moderately dependent (score 9–11), mildly dependent (score 12–19), independent (score 20) [10]; (d) nutritional status based on Mini Nutritional Assessment Full Form: normal (score > 23.5), at risk of malnutrition (score 17–23.5), malnourished (score < 17); (e) number of routinely consumed drugs/medications were obtained from self-report and medical record; (f) comorbidities were obtained from medical records.

Additional baseline data besides the aforementioned were collected from subjects who were selected for the 12-month cohort, including: (a) mental status based on Global Depression Scale – 15 items: normal (score < 5), strong probability of depression (score 5–9), and indicative of depression (score 10 or more); (b) comorbidities based on Cumulative Illness Rating Scale-Geriatric Version (CIRS-G) [11]; (c) QoL; (d) handgrip strength; and (e) usual gait speed.

Frailty state was assessed using the FI-40 questionnaire developed by Mitnitski et al. [12] The frailty state was classified into three groups based on the Frailty Index: frailty index ≤0.08 was classified as robust/fit, > 0.08–< 0.25 as prefrail, and ≥ 0.25 as frail [13]**.** QoL was evaluated based on the general health condition domain of the Short Form-12 (SF-12) questionnaire, which categorized the QoL into excellent, very good, good, fair, and poor [14]**.** The handgrip strength was measured using a handgrip dynamometer (Jamar Hydraulic Hand Dynamometer Model J00105, Lafayette Instrument, Lafayette, Indiana, USA). The examination was conducted according to the American Society of Hand Therapist recommendations. Subjects were asked to sit with elbows flexed in 90° angle, shoulder adducted, and forearm in a neutral position. Subjects were then instructed to inhale a deep breath and to strongly and quickly squeeze the handgrip dynamometer while exhaling. The measurement was taken three times using the dominant hand with 30-min intervals between each measurement and the highest value out of three measurements was recorded [15]**.** In this study we categorized the result into low or normal handgrip strength based on the cut-off value of < 26 kg in men and < 18 kg in women, as defined by the Asian Working Group of Sarcopenia (AWGS) [16]**.** Gait speed was measured using a 15-ft walking test as recommended by the Cardiovascular Health Study. In the test, subjects were instructed to walk with their usual walking pace. Time was recorded from when subjects stepped on the start line to when they stepped on the finish line. Subjects were allowed to pause during the test and the timing was continued until they reached the finish line [17]**.** The time was recorded in seconds. The value of gait speed was calculated by dividing a constant distance of 4.57 m by the time (meter/second). In this study, usual gait speed was classified into slow and normal speed based on the cut-off value of < 0.8 m/s, as defined by AWGS [16]**.**

### Data collection at follow-up

Data collection for the 12-month cohort included a reassessment of frailty state, functional status, nutritional status, cognitive status, comorbidities, number of drugs routinely consumed, QoL, handgrip strength, and gait speed. Frailty state transition was defined as [1] frailty state worsening (e.g., transition of frailty state from robust to prefrail or frail, from prefrail to frail); [2] frailty state improvement (e.g., transition of frailty state from frail to prefrail or robust, from prefrail to robust); or [3] constant frailty state (i.e., no transition of frailty state). In this study, frailty state transition further was categorized into two groups: [1] Frailty state improvement and constant frailty state, and [2] frailty state worsening.

### Statistical analysis

The prevalence of frailty was obtained by calculating the proportions of frailty from the total subjects. Furthermore, bivariate and multivariate analyses were performed for the cross-sectional study. Independent variables were categorized into two groups. The subjects were categorized based on their sex into male or female. Categories according to age group were: [1] < 70 years, and [2] ≥70 years. Categories according to frailty status were: [1] nonfrail (robust and prefrail), and [2] frail. Categories according to nutritional status were: [1] normal, [2] at risk of malnutrition and malnourished. Categories according to functional status were divided into two categories: [1] dependent (for subjects totally dependent, heavily dependent, moderately dependent, and mildly dependent), [2] independent.

The subjects were categorized based on the number of drugs routinely consumed into having polypharmacy (i.e., consumption of five or more drugs) and not having polypharmacy. Bivariate analysis was performed to assess the association between variables and frailty state using a chi-square test. Variables with *p*-value < 0.25 were reanalyzed using multiple logistic regression to identify the risk factors of frailty among those variables. All analyses were performed with SPSS Version 16 (IBM, Armonk, New York, USA). All reported *p*-values were two-sided. *p*-value < 0.05 was considered statistically significant.

Descriptive analysis was performed with all subjects who completed the 12-month follow-up period by calculating the means of numerical variables and the proportions of categorical variables. Furthermore, bivariate and multivariate analyses were performed on data from the 12-month cohort. Independent variables were categorized into two categories: (i) age group (< 70 years, ≥70 years); (ii) gender (male, female); (iii) nutritional status (normal or at risk of malnutrition, malnourished); (iv) functional status categorized into dependent and independent based on Barthel index ADL; (v) mental status (normal or probably depressed, indicative of depression); (vi) QoL was categorized based on clinical judgment as positive QoL (excellent, very good, and good QoL based on SF-12) and negative QoL (fair and poor QoL based on SF-12); (vii) comorbidities (CIRS-G < 5, ≥ 5); (viii) polypharmacy (yes, no); (ix) usual gait speed (slow, normal); and (x) handgrip strength (low, normal). Bivariate analysis was performed using the chi-square test to assess the association between baseline variables and frailty state worsening. Variables with *p*-value < 0.25 were reanalyzed using multiple logistic regression to identify the prognostic factors for frailty state worsening.

The identified prognostic factors were utilized to develop a scoring system to predict frailty state worsening after 12 months. Contribution scores of each identified prognostic factor were calculated by (i) dividing each prognostic factor’s coefficient *B* by its standard error (coefficient *B*/SE = X), (ii) setting the lowest X value as a reference value, and (iii) dividing each X value by the reference value. Performance of the scoring system was evaluated based on the analysis of calibration and discrimination of the scoring system. Calibration, which is the agreement between observed outcomes and predicted risks, was determined by performing the Hosmer–Lemeshow test. Discrimination, which is the ability to distinguish accurately those with and without the outcome, was quantified using the *c*-statistic and described by Areas Under the Curve (AUC) and Receiver Operating Characteristic curves. The *c*-statistic value ranged from 0.5 to 1 and was considered better if it lay closer to 1. Both calibration and discriminating performance were evaluated using the bootstrap resampling method to assess the internal validity. A logistic regression model with backward elimination of predictors was repeated for each of 1000 bootstrap resamplings to obtain the value of the Hosmer–Lemeshow test and *c*-statistic, which showed the maintenance of scoring performance after internal validation.

## Results

### Cross-sectional study

There were 448 subjects recruited at the beginning of the study. The characteristics of those subjects are shown in Table 1. Among those subjects, 13.2% were robust, 61.6% were prefrail, and 25.2% were frail. Most subjects were female (59.8%). The mean age was 72.4 years. The bivariate analysis showed that age (OR 2.83; 95% CI 1.69–4.73), nutritional status (OR 4.55; 95% CI 2.88–7.20), functional status (OR 3.97; 95% CI 2.54–6.20), and polypharmacy (OR 1.82; 95% CI 1.05–3.15) were found to be significantly associated with frailty (*p* < 0.05). The result from the logistic regression analysis is presented in Table 2. It shows that age (OR 2.72; 95% CI 1.58–4.76), functional status (OR 2.89; 95% CI 1.79–4.67), and nutritional status (OR 3.75; 95% CI 2.29–6.13) were identified as the risk factors of frailty.

**Table 1:** Characteristics of subjects (*n* = 448)

Characteristics	*n* (%)
Sex	
Female	268 (59.8)
Male	180 (40.2)
Age group	
60–69 years	158 (35.3)
≥ 70 years	290 (64.7)
Frailty status	
Robust	59 (13.2)
Prefrail	276 (61.6)
Frail	113 (25.2)
Functional status	
Independent	301 (67.2)
Dependent	147 (32.8)
Nutritional status	
Normal	327 (73.0)
Risk of malnutrition	110 (24.6)
Malnourished	11 (2.5)
Polypharmacy	
No	109 (24.3)
Yes	339 (75.7)
Comorbidities	
Hypertension	264 (58.9)
Diabetes Mellitus	168 (37.5)
Coronary heart disease	54 (12.1)
Dyslipidemia	159 (35.5)
Osteoarthritis	142 (31.7)
Chronic kidney disease	69 (15.9)
Cataract	49 (10.9)
Osteoporosis	44 (9.8)

**Table 2:** Risk factors of frailty in Indonesian elderly (*n* = 448)

	Coefficient *B*	Standard error	*P*-value	OR (95% CI)
Age group (≥ 70 years)	1.0	0.3	< 0.001	2.72 (1.58–4.76)
Functional status (dependent)	1.1	0.2	< 0.001	2.89 (1.79–4.67)
Nutritional status (at risk of malnutrition and malnourished)	1.3	0.3	< 0.001	3.75 (2.29–6.13)

### Prospective cohort study

Out of the total 448 subjects, 171 subjects from RSCM were included in the prospective cohort over 12 months. Nine subjects could not be contacted for follow-up due to phone number error or moving to other hospitals for treatment in the 12th-month follow-up, thus only 162 subjects completed the study. The dropout of those nine subjects (5.2%) did not result in discrepancies between baseline characteristics of subjects with or without dropout. Characteristics of subjects (*n* = 162) at baseline and after follow-up are presented in Table 3. Their mean age was 72.9 years. Most of them had normal nutritional (84%) and mental status (92.6%) and were independent (74.1%) at baseline. Most of the subjects were prefrail (70.4%) and only a few were robust (5.6%) at baseline.

**Table 3:** Characteristics of subjects in the prospective cohort over 12 months (*n* = 162)

Characteristics	At Baseline	At 12th Month
Age, mean ± SD, years	72.9 ± 5.9	73.0 ± 5.9
Sex, no. (%)		
Female	93 (57.4)	93 (57.4)
Male	69 (42.6)	69 (42.6)
Functional status, *n* (%)		
Independent (ADL score = 20)	120 (74.1)	100 (61.7)
Dependent (ADL score < 20)	42 (25.9)	62 (38.3)
Nutritional status, *n* (%)		
Normal	136 (84)	131 (80.9)
At Risk of Malnutrition	25 (15.4)	30 (18.5)
Malnourished	1 (0.6)	1 (0.6)
Mental status, *n* (%)		
Normal	150 (92.6)	154 (95.1)
Strong Probability of Depression	9 (5.6)	7 (4.3)
Indicative of Depression	3 (1.9)	1 (0.6)
Frailty state, *n* (%)		
Robust	9 (5.6)	2 (1.2)
Prefrail	114 (70.4)	104 (64.2)
Frail	39 (24.1)	56 (34.6)
Quality of life		
Positive (excellent, very good, and good)	58 (35.8)	58 (35.8)
Negative (fair and poor QoL)	104 (64.2)	104 (64.2)
Commorbidities (CIRS-G Score), mean ± SD	11.8 ± 3.69	10.0 ± 3.5
Number of drugs consumed, mean ± SD	6.9 ± 2.75	6.9 ± 2.7
Handgrip strength, mean ± SD, kg	18.4 ± 6.61	17.1 ± 7.1
Gait speed, mean ± SD, m/s	1.0 ± 0.3	0.8 ± 0.3

The proportion of subjects who were frail increased after 12 months, while few subjects became robust or prefrail. Analysis of the frailty state transition after 12 months of cohort showed that 94 (58.0%) of the subjects had constant frailty state and only 24 (14.8%) had frailty state improvement. Furthermore, 44 (27.2%) of the subjects had frailty state worsening.

Bivariate analysis showed that variables significantly associated with frailty state worsening in 12 months were being 70 years or older (RR 1.36; 95% CI 1.16–1.59, *p* < 0.05), having strong probability of depression or indicative of depression (RR 3.75; 95% CI 1.25–11.21, *p* < 0.05), negative QoL (RR 1.36; 95% CI 1.09–1.68, *p* < 0.05), and slow gait speed (RR 2.68; 95% CI 1.57–4.57, *p* < 0.05) as shown in Table 4. Multivariate analysis of those variables showed that the significant prognostic factors for frailty state worsening were being 70 years or older (OR 3.9; 95% CI 1.2–12.3, *p* < 0.05), negative QoL, i.e., fair and poor QoL (OR 2.5; 95% CI 1.1–5.9, *p* < 0.05), and slow gait speed (OR 2.8; 95% CI 1.3–6.4, *p* < 0.05) as seen in Table 5.

**Table 4:** The association of baseline variables with frailty state worsening (*n* = 162)

Variables	Frailty state transition		*P*-value	RR (95% CI)
	Frailty state worsening [*n* (%)]	Frailty state improvement or constant [*n* (%)]		
Age group				
≥ 70 years	40 (33.6)	79 (66.4)	0.002	1.36 (1.16–1.59)
60–69 years	4 (9.3)	39 (90.7)		
Sex				
Female	26 (28)	67 (72)	0.79	1.04 (0.78–1.39)
Male	18 (26.1)	51 (73.9)		
Nutritional status				
At risk of malnutrition and malnourished	9 (34.6)	17 (65.4)	0.35	1.42 (0.68–2.95)
Normal	35 (25.7)	101 (74.3)		
Functional status (ADL)				
Dependent	14 (33.3)	28 (66.7)	0.29	1.34 (0.78–2.30)
Independent	30 (25.0)	90 (75.0)		
Mental status				
Strong probability of depression and indicative of depression	7 (58.3)	5 (41.7)	0.01	3.75 (1.25–11.21)
Normal	37 (24.7)	113 (75.3)		
Quality of life				
Negative	35 (33.7)	69 (66.3)	0.01	1.36 (1.09–1.68)
Positive	9 (15.5)	49 (84.5)		
Comorbidities (CIRS-G score)				
≥ 5	40 (26.0)	114 (74.0)	0.14	0.94 (0.85–1.04)
< 5	4 (50.0)	4 (50.0)		
Polypharmacy (≥ 5 drugs)				
Yes	39 (29.3)	94 (70.7)	0.18	1.11 (0.96–1.28)
No	5 (17.2)	24 (82.8)		
Gait speed				
Slow (< 0.8 m/s)	19 (50.0)	19 (50.0)	0.00	2.68 (1.57–4.57)
Normal (≥0.8 m/s)	25 (20.2)	99 (79.8)		
Handgrip strength				
Low	34 (31.2)	75 (68.8)	0.09	1.22 (0.98–1.50)
Normal	10 (18.9)	43 (81.1)

**Table 5:** Prognostic factors of 12-month frailty state worsening (*n* = 162)

	Coefficient *B*	Standard error	*P*-value	OR (95% CI)
Age group (≥ 70 years)	1.4	0.6	0.02	3.9 (1.2–12.3)
Negative QoL (Fair or Poor QoL)	0.9	0.4	0.03	2.5 (1.1–5.9)
Slow Gait speed (< 0.8 m/s) Constant	1.0−3.0	0.4	0.01	2.8 (1.3–6.4)

The identified prognostic factors were utilized to develop a prognostic scoring system that predicts frailty state worsening in 12 months and its cut-off value **(**Table 6**)**. A cut-off of ≥2 had 84.1% sensitivity and 57.6% specificity, thus was set as the threshold. This prognostic score showed good calibration (Hosmer–Lemeshow test *p* = 0.11) and good discrimination (AUC value 0.734, 95% CI 0.647–0.820). Internal validation of this score using the bootstrap resampling method showed good maintenance of its performance (Hosmer–Lemeshow test *p* = 0.12).

**Table 6:** Prognostic score for frailty state worsening after 12 months

No.	Variable	Patient Score	
		Yes	No
1	Age ≥ 70 years	1	0
2	Quality of life: Fair or Poor	1	0
3	Gait speed less than 0.8 m/s	1	0
	Total Score	3	

QoL based on general health condition domain in SF-12 questionnaire; Slow gait speed is < 0.8 m/s according to 15-ft walking test; Score ≥ 2 = frailty state worsening; Score < 2 = frailty state improvement

Implementation of the scoring system can be demonstrated by this following case: a 75-year old patient who is prefrail has a gait speed of 0.5 m/s and good QoL based on the general health condition domain in the SF-12 questionnaire. According to the above prognostic score, her score was 2, which means that the patient is predicted to become frail after 12 months.

## Discussion

### Frailty status: prevalence and its risk factors

From this multicenter cross-sectional study among outpatient subjects aged 60 years or above with a mean age of 72.4 years, we found that the prevalence of frailty according to frailty assessment using FI-40 items was 25.2%. This prevalence is comparable with the finding from a study by Tan et al., which found that the prevalence of frailty among Singaporean subjects aged 65 years or above with mean age of 76.6 (SD 6.5) years was 27% [18]. We must bear in mind that the prevalence of frailty depends on the tools used to assess frailty status and the setting (e.g., community-based, hospitalized, or institutionalized). Thus, different tools and different settings may lead to a different result in frailty prevalence. In interpreting the result about the prevalence of frailty in this study in subjects who had received geriatric service in a referral hospital, the prevalence might be different than other Indonesian elderly populations.

Age is one of the risk factors of frailty identified in this study. In this study, we used 70 years as the cut-off to categorize subjects based on age. This cut-off was chosen because the life expectancy of Indonesian elderly is 70.1 years [19]. We would like to compare those who lived beyond the average life expectancy and younger subjects. The risk of frailty was 2.7 times greater among those aged 70 years or above. This result is similar to reports in other studies conducted in Turkey and Brazil, which identified age as a factor significantly associated with frailty [20, 21]. Phenotype of aging manifested due to a discrepancy between stressors and protective mechanisms, as well as failure to compensate, which results in defects. The manifestation of aging phenotype will exacerbate frailty, increase susceptibility to disease, and failure to thrive [22].

Being malnourished or being at risk of malnutrition was identified as a risk factor of frailty in this study. This result is in line with the findings from previous studies by Wei et al. and Eyigor et al. [20, 23] Nutrition has been proven to be an important factor for the development of frailty. Higher protein intake was associated with lower frailty incidence [24]. Nutritional states are also associated with frailty state transition and it is suggested to monitor changes in nutritional status to prevent worsening of the frailty state [20].

In our study, the risk of frailty was 2.9 times greater among elderly with dependent functional status. Similarly, the previous study reported that ADL showed inverted association with frailty. Besides overweight, obesity, and increased comorbidities, physical inactivity is another known risk factor of frailty [25].

### Frailty transition, its prognostic factors, and prognostic scoring system

This cohort study proved that frailty is a dynamic process, even in a relatively short (i.e., 12 months) follow-up period. Our findings were comparable to those with a longer follow-up period [6, 8]. This probably means that the observation of frailty state transition in a short period of follow-up (i.e., 12 months) may reflect frailty state transition in the next following years. The proportion of subjects who experienced frailty state worsening was higher than those who had constant frailty state and frailty state improvement. The percentage of subjects who experienced frailty state worsening was smaller than that in Wei et al. and Trevisan et al. (27.2% vs 46.8% vs 32.7%) [9, 23]. Furthermore, frailty states’ worsening in our study was slightly higher than Alencar’s, which had a similar follow-up period (27.2% vs 24.2%) [7]. Most of our subjects had a constant frailty state (58%). However, frailty state improvement in our study was lower than Faria et al.’s (14.8% vs 45%) [26]. Nevertheless, it showed that there is a possibility for individuals to transition into lesser frailty states, although the probability is low. Thus, effort on frailty state improvement in prefrail and frail older persons should not be neglected.

Unlike previous studies in a community setting [6–9], the definition of frailty state in this prospective cohort study was performed using FI-40, which enabled the collection of new data on the frailty state transition and its prognostic factors. FI-40 is the most suitable instrument to evaluate frailty state in the subjects, especially in a hospital setting. Furthermore, it is the only instrument that covers all of the frailty factors and uses a continuous scoring system [27].

Our study found that the older age group (age ≥ 70 years) had increased risk of frailty state worsening in 12 months. This finding was in line with previous longitudinal studies with a longer follow-up period in European and East Asian populations, which found that older age was associated with the transition into worse frailty state [9, 28]. A previous study in a large Italian population (*n* = 2925) with a mean age of 74.4 ± 7.3 years identified older age as one of the factors associated with frailty state worsening over a mean of the 4.4-year cohort (OR 1.11, 95% CI 1.10–1.12, *p* < 0.001 for frailty state worsening) [9]. In addition, a cohort of 2 years on community-dwelling older Chinese (mean age of 73.5 ± 4.7 years for males and 73.8 ± 5.0 for females) reported that increasing age was one of the factors significantly associated with frailty status exacerbation [28]. Therefore, our finding supports the existing evidence that older age, in this case being 70 years or older, increases the risk of frailty status worsening within a follow-up period as short as 12 months.

The correlation between frailty and HR-QoL in older adults has been extensively studied [5, 6, 29, 30]. Prior study of QoL and frailty revealed that prefrail and frail subjects presented with worse physical and cognitive HR-QoL compared with fit subjects [4]. A previous longitudinal study by Gobbens et al. has shown the ability of frailty assessment in predicting the presence of adverse outcomes including poor QoL in 1–2 years [31]. However, the possibility of QoL in affecting frailty state transition of older adults has scarcely been explored. In our study, the prevalence of subjects with negative QoL (i.e., fair and poor QoL) was 64.2% based on general health domain in the SF-12 questionnaire and those who had a higher risk of experiencing frailty state worsening in 12 months compared with subjects with better QoL (RR 2.5; 95% CI 1.1–5.9). The finding from our prospective observation suggested that there was a clear time relationship between the QoL and frailty state worsening, in which negative QoL occurred before frailty state worsening. A longitudinal study of psychological well-being and frailty found that better psychological well-being based on the CASP-19 QoL questionnaire was protective against being prefrail (RR 0.79; 95% CI 0.71–0.89) and being frail (RR 0.62; 95% CI 0.52–0.74) at 4-year follow-up [32]. The confidence interval in our study was wide compared to those studies with larger number of subjects, which could be explained by the smaller sample size in our study.

Our study also emphasized the essential role of functional mobility in predicting the frailty state transition. Our study found that subjects with slower usual gait speed (gait speed < 0.8 m/s based on 15-ft walking test) had an increased risk of experiencing frailty state worsening in 12 months. There are two perspectives of assessing frailty: frailty as deficit accumulation and frailty as a phenotype. Our study used the FI-40 questionnaire to assess frailty status as deficits accumulation. The FI-40 did not incorporate the gait speed component in the measurement. Thus, to complement the data on frailty, we also measured subjects’ gait speed, which represents physical frailty components. The results of this study clearly reaffirm that slow gait speed is closely related to frailty, especially the worsening frailty state transition. Walking requires the coordination of various organ systems and consumes energy, thus decreased organ function and increased energy consumption for walking may be reflected through slowing gait speed. Slowing gait speed can be caused by the presence of comorbidities (e.g., cardiovascular disease and musculoskeletal problems) and frailty [33]. Gait speed can be used to assess multiple organ systems simply and comprehensively [34]. Numerous prior studies have reported the association between gait speed and frailty. A study by Jung et al. in rural community-dwelling Korean elderly population reported that slower gait speed was associated with worse frailty status [35]. Furthermore, a cross-sectional study in Malaysia by Badrasawi et al. reported that slower rapid pace gait speed (> 5.2 s) which reflected poor physical function was one of the significant risk factors of frailty [36]. It was argued that lower rapid pace gait speed had high correlation with slowness because gait speed was one of the criteria that define frailty, besides the four other criteria in Fried criteria [36]. While the study in the Malaysian population had identified lower rapid gait speed as one of the risk factors of frailty, our study identified lower usual gait speed (gait speed < 0.8 m/s based on 15-ft walking test) as predictors of frailty state worsening in 12 months.

Similar to our study, the previous study by Fallah et al. found that mobility was significantly associated with frailty state transition in 18 months [37]. It is interesting to notice the different cut-off values for gait speed between our studies. In the present study, subjects were categorized as having lower gait speed when 15-ft walking test results were less than 0.8 m/s, while in their study gait speed less than 0.6 m/s based on a 20-ft walking test was considered slow. The follow-up periods in the studies were both relatively short (12 and 18 months). We can infer from both studies that gait speed can be used to predict frailty status transition in the relatively near future. Another longitudinal study over a 12-month cohort in Malaysian older adults reported that walking was the only type of physical activity according to the PASE questionnaire that was significantly associated with frailty state transition [38].

To our knowledge, the present study is the first multicenter cross-sectional and longitudinal study on frailty in an outpatient setting. Conducting this study in an outpatient setting enabled frailty state evaluation to be performed comprehensively using FI-40 as the instrument, which had seldom been done in previous community-setting studies [6–9]. Furthermore, we developed a scoring system to identify older patients who have a higher risk of frailty state worsening in 12 months, which can be easily applied by healthcare professionals. Our prognostic study was the first one to develop a prediction model for frailty transition in Indonesia. We applied mathematical models to predict the phenomenon of frailty transition within 12 months. This simple scoring system consisted of three identified prognostic factors (i.e., functional mobility, QoL, and age), which can be assessed in a short duration of time by asking older patients to complete the 15-ft walking test, interviewing them using the general health domain in the SF-12 questionnaire and recording their age. It should be noted that although the association between gait speed and frailty has been clearly established, gait speed alone should not be used independently to predict frailty transition. Besides the emphasis on gait speed, our study also emphasizes the importance of maintaining good QoL and functional mobility in older adults, especially the prefrail and frail ones. Thus, our prognostic study has combined gait speed, age, and QoL to develop a prognostic model that can be used to predict frailty transition in the 12-month period. Because frailty has been known to be associated with poor health-related outcomes, information about the risk of being frail in the future is important for elderly patients and their families as well as health professionals caring for elderly patients. The development of such prognostic model is important to help physicians and their elderly patients in making a well-informed clinical decision related to high-risk procedures, invasive treatment, or diagnostic tests in particular. Our prognostic model has been proven to have good internal validity, however, future study is needed to assess the external validation of this prognostic model in other Indonesian populations.

However, there were some limitations in our study. Firstly, results from the cross-sectional study could not be used to establish causal relationships between frailty and the independent variables. Secondly, the number of drop out subject in prospective cohort study was 5.2%. Thirdly, because the prospective cohort study took place in RSCM, which is the National Referral Hospital, it resulted in the selection of subjects living in a metropolitan area. Thus, the results may not be generalized to other Indonesian elderly living in rural areas.

## Conclusions

The prevalence of frailty in this study among Indonesian elderly in an outpatient setting was 25.2%. This study identified that frailty was associated with age, functional status, and nutritional status. The identified prognostic factors for frailty state worsening in 12 months were being 70 years or older, having a fair or poor QoL based on the general health domain of the SF-12 questionnaire, and slow gait speed (i.e., < 0.8 m/s based on 15-ft walking test). A prognostic score for frailty state worsening in 12 months was developed based on the identified prognostic factors and showed good performance after internal validation. Further research to evaluate external validation is required to determine the performance of this score in other populations.

## Data Availability

The datasets used and/or analysed during the current study are available from the corresponding author on reasonable request.

## Abbreviations

ADL: Activity of daily living
AUC: Areas under the curves
AWGS: Asian Working Group of Sarcopenia
CIRS-G: Cumulative illness rating scale geriatric
FI-40: Frailty index-40 item
QoL: Quality of life
